# Correlation of Performance Status and Neutrophil-Lymphocyte Ratio with Efficacy in Radioiodine-Refractory Differentiated Thyroid Cancer Treated with Lenvatinib

**DOI:** 10.1089/thy.2020.0779

**Published:** 2021-08-03

**Authors:** Matthew H. Taylor, Shunji Takahashi, Jaume Capdevila, Makoto Tahara, Sophie Leboulleux, Naomi Kiyota, Corina E. Dutcus, Ran Xie, Bruce Robinson, Steven Sherman, Mouhammed Amir Habra, Rossella Elisei, Lori J. Wirth

**Affiliations:** ^1^ Earle A. Chiles Research Institute, Providence Portland Medical Center, Portland, Oregon, USA.; ^2^ Department of Medical Oncology, The Cancer Institute Hospital of JFCR, Tokyo, Japan.; ^3^ Medical Oncology Department, University Hospital Vall d'Hebron, Vall d'Hebron Institute of Oncology (VHIO), Barcelona, Spain.; ^4^ Division of Head and Neck Medical Oncology, National Cancer Center Hospital East, Kashiwa, Japan.; ^5^ Department of Nuclear Medicine and Endocrine Oncology, Gustave Roussy, Villejuif, France.; ^6^ Department of Medical Oncology and Hematology and Cancer Center, Kobe University Hospital, Kusunoki Cho, Chuo-ku, Japan.; ^7^ Eisai Inc., Woodcliff Lake, New Jersey, USA.; ^8^ Department of Biostatistics, Eisai Inc., Woodcliff Lake, New Jersey, USA.; ^9^ Royal North Shore Hospital, University of Sydney, Australia.; ^10^ Department of Endocrine Neoplasia and Hormonal Disorders, Division of Internal Medicine, University of Texas M.D. Anderson Cancer Center, Houston, Texas, USA.; ^11^ Endocrine Unit, Department of Clinical and Experimental Medicine, University of Pisa, Pisa, Italy.; ^12^ Department of Medicine, Massachusetts General Hospital Cancer Center, Boston, Massachusetts, USA.

**Keywords:** differentiated thyroid cancer, ECOG, lenvatinib, NLR, tumor size

## Abstract

***Background:*** Radioiodine-refractory differentiated thyroid cancer (RR-DTC) has a low 10-year patient-survival rate and is challenging to treat. Lenvatinib is a multikinase inhibitor approved for the treatment of RR-DTC. This study aims to assess Eastern Cooperative Oncology Group performance status (ECOG PS) and neutrophil-to-lymphocyte ratio (NLR) as prognostic markers for patients with RR-DTC treated with lenvatinib.

***Methods:*** In this retrospective analysis of the **S**tudy of (**E**7080) **LE**nvatinib in Differentiated **C**ancer of the **T**hyroid (SELECT), patients randomly assigned to receive lenvatinib were classified according to baseline ECOG PS (0 or 1) or baseline NLR (≤3 or >3). The effects of baseline ECOG PS and NLR on progression-free survival (PFS), overall survival (OS), and objective response rate (ORR) were evaluated. In addition, the effects of baseline ECOG PS on the change in diameter of target lesions and correlations between baseline NLR and the sums of the diameters of target lesions were calculated.

***Results:*** Among patients who received lenvatinib, patients with a baseline ECOG PS of 0 had statistically improved PFS (hazard ratio [HR] 0.52; 95% confidence interval [CI 0.35–0.77]; *p* = 0.001), OS (HR 0.42 [CI 0.26–0.69]; *p* = 0.0004), and ORR (odds ratio [OR] 3.51 [CI 2.02–6.10]; *p* < 0.0001) compared with patients with a baseline ECOG PS of 1. Patients who received lenvatinib with a baseline NLR ≤3 also had improved PFS (HR 0.43 [CI 0.29–0.65]; *p* < 0.0001) and OS (HR 0.48 [CI 0.29–0.78]; *p* = 0.0029) versus patients with a baseline NLR >3. Moreover, patients with a baseline NLR ≤3 had a trend toward increased ORR (OR 1.57 [CI 0.94–2.64]; *p* = 0.08) compared with patients with a baseline NLR >3. Treatment-emergent adverse events were generally similar among patients who received lenvatinib, irrespective of patients' ECOG PS at baseline.

***Conclusion:*** Lower ECOG PS and NLR may provide prognostic value for improved efficacy in patients with RR-DTC. ClinicalTrials.gov no. NCT01321554.

## Introduction

The incidence of cancer with thyroid as the site of origin has increased from 3.6 cases per 100,000 in 1973 to 8.7 cases per 100,000 in 2002, representing a 2.4-fold increase in the number of cases diagnosed/year (1). This increase was almost entirely attributable to an increase in papillary thyroid cancers (2), and because the incidence of tumors of ∼2–4 cm in diameter remained stable, this increase was thought to be due to more frequent diagnosis of subclinical tumors as a result of medical imaging (3). However, rates are also increasing for larger thyroid tumors and those with regional and distant spread (4); as such, thyroid cancer mortality has increased by 1.1% per year from 1994 to 2013 (5). Although most patients' differentiated thyroid cancer (DTC) can be cured with surgery, often followed by radioiodine therapy and thyroid-stimulating-hormone suppression, patients with unresectable locally recurrent and/or metastatic radioiodine-refractory (RR)-DTC have a 10-year survival rate of only 19% (6,7).

Lenvatinib is a tyrosine kinase inhibitor of vascular endothelial growth factor receptors 1–3, fibroblast growth factor receptors 1–4, platelet-derived growth factor receptor α, RET, and KIT (8–11). Lenvatinib was approved for the treatment of patients with locally recurrent or metastatic, progressive RR-DTC, based on the results of the phase 3 **S**tudy of (**E**7080) **LE**nvatinib in Differentiated **C**ancer of the **T**hyroid (SELECT) (12,13). In SELECT, median progression-free survival (PFS) was 18.3 months with lenvatinib compared with 3.6 months with placebo. Further analyses of SELECT have indicated that the efficacy and safety of lenvatinib is maintained across several categories of radioiodine refractoriness (i.e., when patients were stratified by RR inclusion criteria: including no radioiodine uptake, disease progression within 12 months of radioiodine therapy, and extensive cumulative radioiodine exposure) (14).

Despite this advancement in treatment, few prognostic factors have been described for RR-DTC. Eastern Cooperative Oncology Group performance status (ECOG PS)—a comprehensive measure of a patient's level of functioning (15)—is associated with survival and response to therapy in patients with multiple types of malignancies, including non-small cell lung cancer and esophageal cancer (16,17). Similarly, elevated neutrophil-to-lymphocyte ratio (NLR) is associated with more aggressive disease and decreased survival in patients with numerous types of solid tumors, including DTC (18,19). When NLR was retrospectively analyzed in patients specifically with RR-DTC treated with lenvatinib in a real-world setting, median overall survival (OS) was significantly longer in patients with lower baseline NLR (20). The mechanism by which elevated NLR is associated with more aggressive disease is not definitively known but may, in part, be explained by tumor-promoting consequences of inflammation (21). Moreover, results of a retrospective review of clinical records indicated that high baseline tumor burden was associated with a worse prognosis in patients with RR-DTC (22). Evidence has shown that treatment-emergent hypertension in patients receiving lenvatinib is associated with longer OS (23), but this correlation is not useful in making decisions regarding when to initiate lenvatinib treatment.

Treatment with tyrosine kinase inhibitors in RR-DTC is not curative and is associated with treatment-emergent adverse events (TEAEs); therefore, identification of prognostic markers could help physicians make the challenging decision of whether to initiate systemic therapy. This exploratory *post hoc* analysis assessed baseline ECOG PS and NLR as potential prognostic indicators in patients with RR-DTC treated with lenvatinib.

## Methods

### Patients

SELECT was a phase 3 randomized, double-blind, multicenter study that compared lenvatinib versus placebo in patients with RR-DTC. Full details of the study have been published (13). A brief summary of eligibility criteria can be found in the Supplementary Material S1. This analysis focuses on patients initially randomized to the lenvatinib arm of the study.

All patients provided written informed consent, and the study protocol was approved by all relevant institutional review bodies. The study was conducted in accordance with the provisions of the Declaration of Helsinki and local laws.

### Study design

In SELECT, patients were randomly assigned (2:1) to receive oral lenvatinib at 24 mg/day or placebo in 28-day cycles (13). Tumor responses were assessed by an independent centralized imaging laboratory using Response Evaluation Criteria In Solid Tumors version 1.1 (RECIST v1.1) every 8 weeks during the randomization phase and every 12 weeks during the extension phase; they were confirmed by an independent imaging review (13). Patients initially randomly assigned to receive placebo were offered open-label lenvatinib on centrally confirmed disease progression. Primary and secondary objectives of SELECT have been previously reported (13). Adverse events (AEs) were categorized according to the National Cancer Institute Common Terminology Criteria for Adverse Events, version 4.0.

### *Post hoc* subgroup analyses and statistical methods

This exploratory *post hoc* subgroup analysis included patients from SELECT who were randomly assigned to receive lenvatinib, using the data cutoff date from the primary analysis (November 15, 2013). Patients were then divided into subgroups according to baseline ECOG PS (0 or 1) and baseline NLR of ≤3 or >3 (the median baseline NLR value for patients receiving lenvatinib was 3.1, thus a cutoff NLR value of 3 was utilized for this analysis). NLR was calculated based on peripheral blood cell counts. As corticosteroid treatment can impact NLR values, a separate analysis was conducted excluding 20 patients who were receiving concomitant corticosteroids at baseline to confirm the accuracy of the NLR data.

Efficacy analyses were conducted with patients who received lenvatinib and were grouped by either ECOG PS or NLR. The effects of baseline ECOG PS and baseline NLR on PFS, OS, and objective response rate (ORR) were evaluated separately. In addition, the effects of baseline ECOG PS on the change in the sums of the diameters of target lesions and correlations between baseline NLR and the sums of the diameters of target lesions were calculated. The number of metastatic sites involved was assessed in patients with a baseline ECOG PS 0 versus 1, as well as NLR ≤3 versus NLR >3. ECOG PS was also measured in patients with a baseline NLR ≤3 and NLR >3. Kaplan–Meier analyses for OS and PFS were also conducted on data from patients who had received placebo, grouped by either ECOG PS or NLR. These results are reported in the Supplementary Material S1.

Statistical analyses were performed using SAS (SAS Institute Inc.) version 9.4. PFS and OS were summarized by using Kaplan–Meier estimates; 95% confidence intervals (CIs) of the median were constructed with a generalized Brookmeyer and Crowley method; hazard ratios (HRs) between subgroups were estimated from an unstratified Cox proportional hazard model; and *p*-values were based on an unstratified log-rank test. The CIs for ORR were calculated by using asymptotic normal approximation; the odds ratios (OR) between subgroups and *p*-values were calculated by using the Chi-square test. Percentage changes from baseline to postbaseline nadir in the sums of diameters of target lesions and percentage changes over time according to ECOG PS at baseline (0 or 1) were plotted and assessed.

Multivariate analyses, which included ECOG PS, NLR, sums of the diameters of target lesions at baseline, and age, were conducted to determine whether any factors had a significant association with OS or PFS. Patients randomly assigned to lenvatinib who had a baseline ECOG PS of 0 or 1 and a nonmissing baseline NLR were included in these analyses. Associations with PFS and OS were based on maximum likelihood estimates for the covariates in the unstratified Cox proportional hazard model. Extent of correlation (negligible, low, etc.) is described as per Hinkle *et al* (24).

Given the *post hoc* nature of the analyses in this article, all reported *p*-values (including those for multivariate analyses) should be considered nominal.

## Results

### Patients

The baseline demographic and disease characteristics of patients from SELECT who were randomly assigned to receive lenvatinib were analyzed according to patients' ECOG PS and are shown in Table 1. Of the 261 patients in the lenvatinib arm, 144 (55.2%) had a baseline ECOG PS of 0 and 104 (39.8%) had a baseline ECOG PS of 1. Demographics and disease characteristics were generally similar among the two ECOG PS groups (Table 1). In addition, more patients with an ECOG PS of 1 had ≥4 metastatic sites (19.2%) by RECIST v1.1 compared with patients with an ECOG PS of 0 (9.0%).

**Table 1.** Baseline Demographic and Disease Characteristics of Patients Randomly Assigned to Receive Lenvatinib, According to Eastern Cooperative Oncology Group Performance Status

Parameter	Baseline ECOG PS 0 (*n* = 144)	Baseline ECOG PS 1 (*n* = 104)
Age, median, years (range)	63.5 (30–80)	63 (27–89)
Age group, *n* (%)		
≤65 years	86 (59.7)	64 (61.5)
>65 years	58 (40.3)	40 (38.5)
Weight, *n* (%)		
<60 kg	33 (22.9)	22 (21.2)
≥60 kg	111 (77.1)	82 (78.8)
Sum of target lesion diameters at baseline, median (mm)	50.1	66.1
No. of metastatic sites, *n* (%)		
0	3 (2.1)	1 (1.0)
1	45 (31.3)	17 (16.3)
2	46 (31.9)	38 (36.5)
3	37 (25.7)	28 (26.9)
≥4	13 (9.0)	20 (19.2)
NLR level, *n* (%)		
≤3	76 (52.8)	44 (42.3)
>3	68 (47.2)	60 (57.7)
Absolute neutrophil count, 10^9^/L, mean (SD)	4.0 (1.7)	4.5 (2.1)
Absolute lymphocyte count, 10^9^/L, mean (SD)	1.3 (0.5)	1.3 (0.6)
Hypertension, *n* (%)		
Yes	81 (56.3)	57 (54.8)
No	63 (43.8)	47 (45.2)
Diabetes, *n* (%)		
Yes	22 (15.3)	14 (13.5)
No	122 (84.7)	90 (86.5)
Proteinuria, *n* (%)		
Positive	5 (3.5)	4 (3.8)
Negative	139 (96.5)	100 (96.2)
Renal impairment,^a^*n* (%)		
Yes	17 (11.8)	10 (9.6)
No	127 (88.2)	94 (90.4)
Hepatic impairment,^b^*n* (%)		
Yes	19 (13.2)	8 (7.7)
Mild	17 (11.8)	8 (7.7)
Moderate	2 (1.4)	0
No	125 (86.8)	96 (92.3)

These data include only patients with baseline ECOG PS values of 0 or 1.

^a^ Defined as baseline serum creatine clearance <60 mL/min.

^b^ Defined as a condition with Common Terminology Criteria for Adverse Events grade ≥1 for any of the following parameters: aspartate aminotransferase, alanine aminotransferase, and bilirubin at baseline; impairment is defined as mild if any parameter is grade 1; impairment is defined as moderate if any parameter is grade 2.

ECOG PS, Eastern Cooperative Oncology Group performance status; NLR, neutrophil-to-lymphocyte ratio; SD, standard deviation.

Patients in the lenvatinib arm were also analyzed according to NLR. A baseline NLR ≤3 was recorded for 121 (46.4%) patients, and 140 (53.6%) patients had a baseline NLR >3; the median baseline NLR for patients randomly assigned to receive lenvatinib was 3.1 (range: 0.7–36.3). Absolute neutrophil and lymphocyte counts by NLR are reported in Supplementary Table S1. Among patients with an NLR ≤3, 62.8% had an ECOG PS of 0 and 37.2% had an ECOG PS ≥1; and among patients with an NLR >3, 48.6% had an ECOG PS of 0 and 51.4% had an ECOG PS ≥1. There was no significant association between baseline NLR and baseline ECOG PS (*p* = 0.2414). The baseline numbers of metastatic sites analyzed according to NLR for patients randomly assigned to receive lenvatinib are shown in Supplementary Figure S1. There was a significant association between higher baseline NLR and presence of ≥3 metastatic sites (*p* = 0.0011). In general, no significant correlations were observed between age and NLR or age and ECOG PS.

### Efficacy

#### Subgroup analysis by baseline ECOG PS (0 or 1) in patients in the lenvatinib arm

**FIG. 1.**
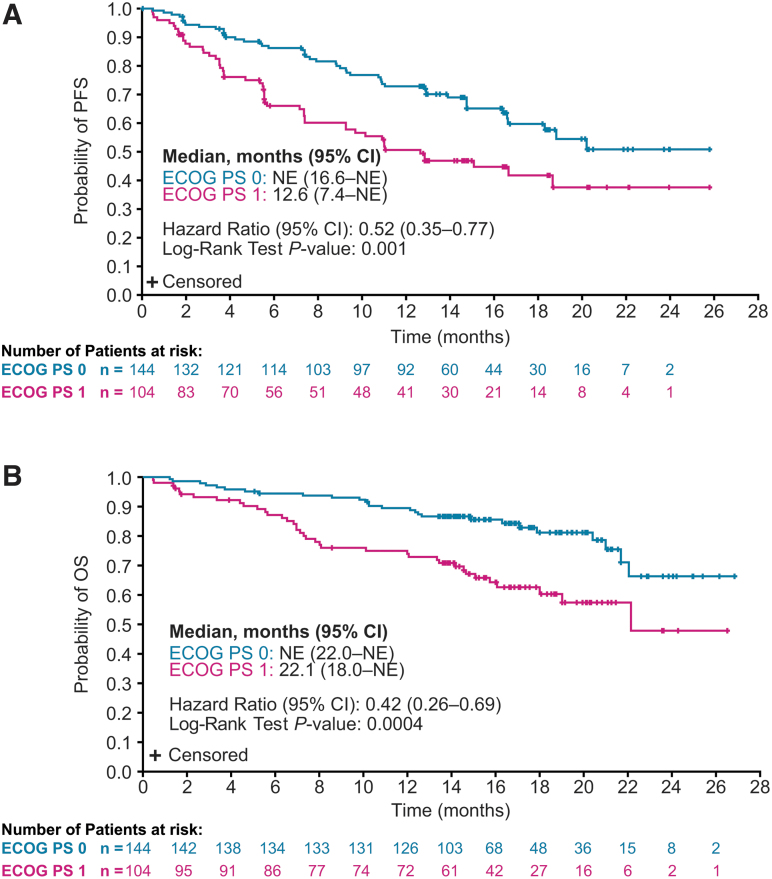
Kaplan–Meier plots of PFS (**A**) and OS (**B**) in patients randomly assigned to receive lenvatinib, and with a baseline ECOG PS of 0 or 1. OS and PFS medians were calculated by Kaplan–Meier estimates, and the corresponding CIs were calculated with a generalized Brookmeyer and Crowley method. The HRs were estimated from an unstratified Cox proportional hazard model, and *p*-values were based on an unstratified log-rank test. CI, 95% confidence interval; ECOG PS, Eastern Cooperative Oncology Group performance status; HR, hazard ratio; NE, not estimable; OS, overall survival; PFS, progression-free survival.

Among patients randomly assigned to lenvatinib, PFS was prolonged for those with a baseline ECOG PS of 0 compared with patients with a baseline ECOG PS of 1 (HR 0.52 [CI 0.35–0.77]; *p* = 0.001) (Fig. 1A). Similar results for the ECOG PS 0 and 1 groups were also observed for OS (HR 0.42 [CI 0.26–0.69]; *p* = 0.0004) (Fig. 1B). Tumor responses by baseline ECOG PS are summarized in Table 2.

**Table 2.** Tumor Responses by Baseline Eastern Cooperative Oncology Group Performance Status in Patients Randomly Assigned to Receive Lenvatinib, as Assessed by Independent Imaging Review Using Response Evaluation Criteria In Solid Tumors Version 1.1

Parameter	ECOG PS 0 (*n* = 144)	ECOG PS 1 (*n* = 104)
Best overall response, *n* (%)		
Complete response	3 (2.1)	1 (1.0)
Partial response	110 (76.4)	52 (50.0)
Stable disease	21 (14.6)	32 (30.8)
Durable stable disease^a^	15 (10.4)	20 (19.2)
Progressive disease	7 (4.9)	10 (9.6)
Unknown	3 (2.1)	9 (8.7)
Objective response rate, *n* (%)	113 (78.5)	53 (51.0)
[CI]	[71.8–85.2]	[41.4–60.6]
Difference, % [CI]	27.5 [15.8–39.2]	
Odds ratio^b^ [CI]	3.51 [2.02–6.10]	
*p*	<0.0001	

^a^ Stable disease with a duration of ≥23 weeks.

^b^ The odds ratio and *p*-value were calculated by using the Chi-square test, and corresponding CIs were generated based on asymptotic normal approximation.

CI, 95% confidence interval.

**FIG. 2.**
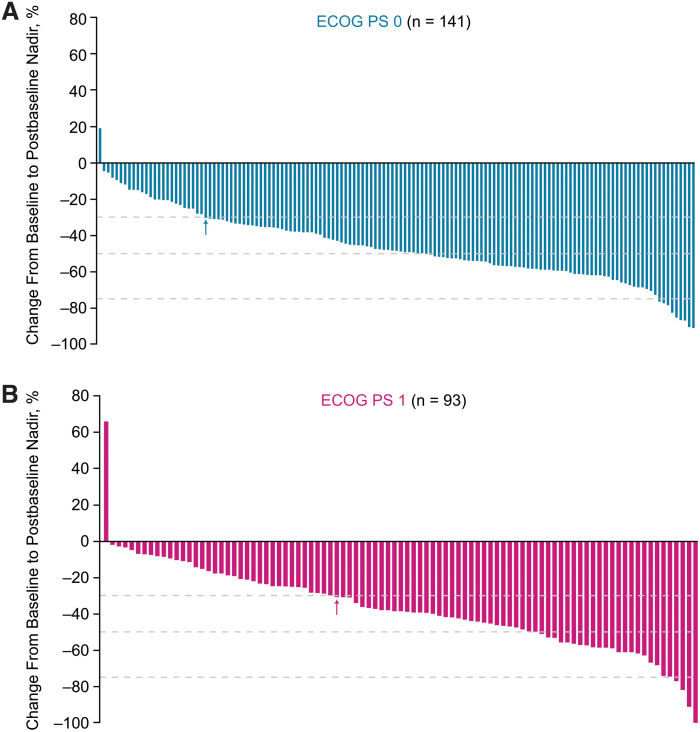
Percentage change from baseline to postbaseline nadir in the sums of diameters of target lesions in patients randomly assigned to receive lenvatinib, and with a baseline ECOG PS of 0 (**A**) or 1 (**B**). *n* = the number of patients with both baseline and at least 1 postbaseline target lesion assessment. Arrows indicate the 30% threshold for partial response, according to RECIST version 1.1. RECIST, Response Evaluation Criteria In Solid Tumors.

The ORR in patients with an ECOG PS of 0 was 78.5% [CI 71.8–85.2] versus 51.0% [CI 41.4–60.6] in patients with an ECOG PS of 1 (OR 3.51 [CI 2.02–6.10]; *p* < 0.0001). Percent changes in the sums of diameters of target lesions from baseline to postbaseline nadir according to baseline ECOG PS are shown in Figure 2. Mean maximum percent decrease in tumor size was numerically greater in patients with a baseline ECOG PS of 0 (−46.1%) compared with patients with a baseline ECOG PS of 1 (−37.2%) (Supplementary Fig. S2A). The shrinkage in the sums of diameters of target lesions increased over time, irrespective of patients' ECOG PS at baseline (Supplementary Fig. S2B).

#### Subgroup analysis by baseline NLR in patients in the lenvatinib arm

**FIG. 3.**
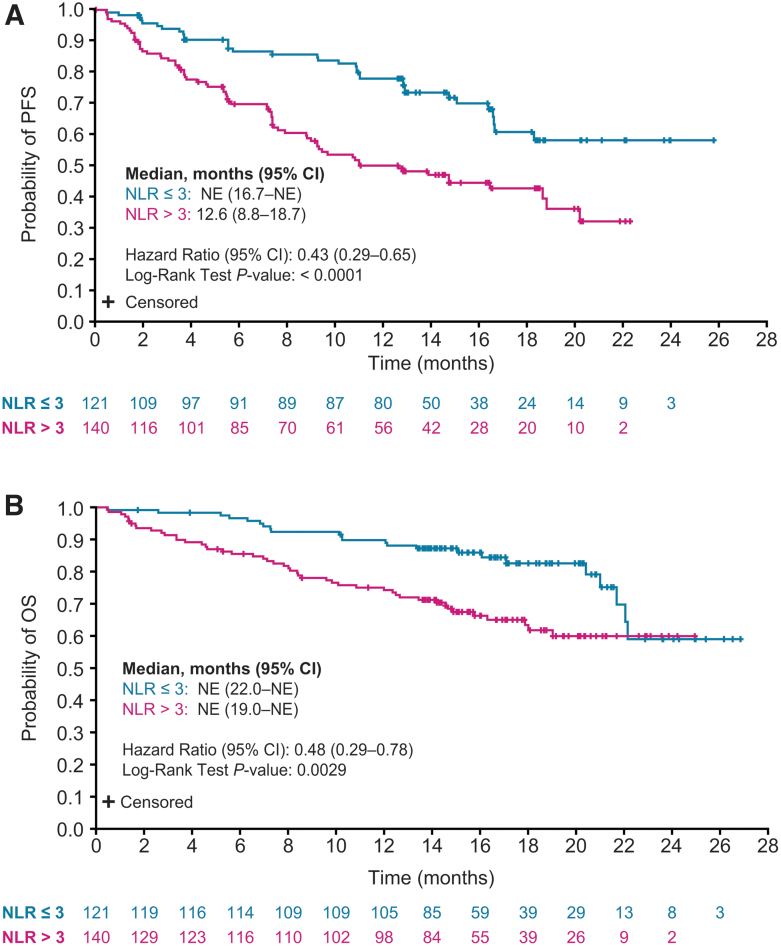
Kaplan–Meier plots of PFS (**A**) and OS (**B**) in patients randomly assigned to receive lenvatinib by NLR (≤3 or >3). OS and PFS medians were calculated by Kaplan–Meier estimates, and the corresponding 95% CIs were calculated with a generalized Brookmeyer and Crowley method. The HRs were estimated from an unstratified Cox proportional hazard model, and *p*-values were based on an unstratified log-rank test. NLR, neutrophil-to-lymphocyte ratio.

PFS was prolonged in patients with an NLR ≤3 versus patients with an NLR >3 (HR 0.43 [CI 0.29–0.65]; *p* < 0.0001) (Fig. 3A). Patients with an NLR ≤3 also had improved OS compared with patients with an NLR >3 (HR 0.48 [CI 0.29–0.78]; *p* = 0.0029) (Fig. 3B). When patients receiving concomitant corticosteroids at baseline (*n* = 20, 7.7%) were excluded from analysis, median PFS and OS values by baseline NLR were similar to those of the overall lenvatinib patient population (Supplementary Fig. S3).

**FIG. 4.**
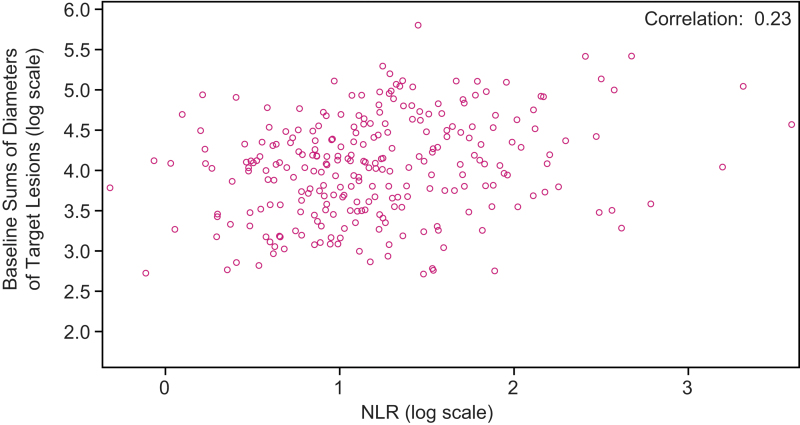
Correlation between baseline sums of diameters of target lesions and NLR in patients randomly assigned to receive lenvatinib (log scales).

ORR was 70.2% [CI 62.1–78.4] for patients with an NLR ≤3 and 60.0% [CI 51.9–68.1] for patients with an NLR >3 (OR 1.57 [CI 0.94–2.64]; *p* = 0.08). Further, among patients without concomitant corticosteroid use at baseline, patients with an NLR ≤3 (*n* = 116) had an ORR of 71.6% [CI 63.3–79.8], and patients with an NLR >3 (*n* = 125) had an ORR of 63.2% [CI 54.8–71.7] (OR 1.46 [CI: 0.85–2.52]; *p* = 0.17) (Supplementary Table S2). Overall, the correlation (0.23) between sums of diameters of target lesions at baseline and NLR was negligible (Fig. 4).

#### Results of the multivariate analyses, which included ECOG PS, sums of diameters of target lesions, NLR, and age at baseline

According to multivariate analyses, baseline ECOG PS (0 vs. 1) had a significant association with PFS (*p* = 0.0032) and OS (*p* = 0.0012) (Table 3). The sums of diameters of target lesions at baseline approached a significant association with PFS (*p* = 0.0587), but was not significantly associated with OS (*p* = 0.4145). Baseline NLR was significantly associated with PFS (*p* = 0.0009) and OS (*p* = 0.0128). Patient age at baseline was significantly associated with PFS (*p* = 0.0459), but not with OS (*p* = 0.2125).

**Table 3.** Multivariate Analysis of Efficacy Endpoints with Baseline Characteristics in Patients Randomly Assigned to Receive Lenvatinib

Baseline efficacy endpoints	*p*-value	Hazard ratio [CI]
Progression-free survival		
ECOG PS (0 vs. 1)	0.0032	0.55 [0.36–0.82]
Sums of target lesions	0.0587	1.00 [1.00–1.01]
NLR	0.0009	1.08 [1.03–1.12]
Age	0.0459	1.02 [1.00–1.04]
Overall survival		
ECOG PS (0 vs. 1)	0.0012	0.43 [0.26–0.72]
Sums of target lesions	0.4145	1.00 [1.00–1.01]
NLR	0.0128	1.07 [1.02–1.14]
Age	0.2125	1.02 [0.99–1.04]

#### Subgroup analyses by baseline ECOG PS (0 or 1) and by baseline NLR in the placebo arm

PFS and OS data by baseline ECOG PS for patients who received placebo are provided in Supplementary Figure S4. Similar data by baseline NLR for patients who received placebo are provided in Supplementary Figure S5.

### Safety

The median duration of treatment with lenvatinib was 14.3 months (range: 0.8–26.8) for patients with a baseline ECOG PS of 0 and 10.6 months (range: 0.2–26.5) for patients with a baseline ECOG PS of 1. The median dose intensity of lenvatinib was 16.2 mg/day/patient (range: 5.8–24.0) for patients with an ECOG PS of 0 at baseline and 17.6 mg/day/patient (range: 6.5–24.0) for patients with an ECOG PS of 1 at baseline.

An overview of TEAEs and the most common TEAEs by baseline ECOG PS are summarized in Supplementary Table S3. Any-grade TEAEs occurred in nearly all patients who received lenvatinib, irrespective of ECOG PS at baseline (ECOG PS 0, TEAEs in 100%; ECOG PS 1, TEAEs in 99%). TEAEs led to lenvatinib discontinuation in 11.1% of patients with an ECOG PS of 0 and in 19.2% of patients with an ECOG PS of 1; 73.6% of patients with an ECOG PS of 0 experienced a TEAE resulting in lenvatinib dose reduction, and 59.6% of patients with an ECOG PS of 1 had a TEAE resulting in dose reduction. TEAEs led to lenvatinib interruption in 81.9% of patients with an ECOG PS of 0 and in 82.7% of patients with an ECOG PS of 1. The most common TEAEs (data presented as ECOG PS 0/ECOG PS 1) were diarrhea (76.4%/53.8%), hypertension (72.2%/66.3%), decreased appetite (54.2%/51.9%), decreased weight (54.2%/47.1%), and nausea (46.5%/45.2%). Patients with an ECOG PS of 0 had fewer serious TEAEs (41.0%) than did patients with an ECOG PS of 1 (60.6%). Most patients experienced a treatment-related AE, irrespective of baseline ECOG PS (ECOG PS 0, 98.6%; ECOG PS 1, 95.2%). Percentages of patients who experienced treatment-related AEs of grade ≥3 were also similar between the ECOG PS groups (ECOG PS 0, 77.8%; ECOG PS 1, 72.1%).

## Discussion

In SELECT, lenvatinib demonstrated a significant PFS benefit in patients with RR-DTC (13). This exploratory *post hoc* analysis of SELECT provides insights into markers of efficacy among patients with RR-DTC treated with lenvatinib. Specifically, patients with lower ECOG PS and lower NLR (largely driven by lower absolute neutrophil counts; Table 1 and Supplementary Table S1) at baseline had improved PFS, OS, and ORR.

The results of this *post hoc* analysis suggest that initiating treatment before a worsening in ECOG PS may improve clinical outcomes. Similarly, patients who are more fit and indicated for systemic therapy may benefit more from earlier treatment with lenvatinib. As expected, the sum of target lesion diameters at baseline was less in patients treated with lenvatinib with an ECOG PS of 0 compared with that measured in patients with an ECOG PS of 1 (Table 1). Nonetheless, patients with an ECOG PS of 0 experienced a higher ORR and greater percentage decrease in the sums of the diameters of target lesions, which was maintained over time (Supplementary Fig. S2). Patients with lower ECOG PS and NLR (regardless of treatment) also had improved survival outcomes (Figs. 1 and 3 and Supplementary Figs. S4 and S5). In a separate analysis of SELECT with updated data, durations of response with lenvatinib were shorter in patients with increased tumor burden (25). In combination with our analysis, these cumulative data suggest that, regardless of the disease-state marker, outcomes are improved when treatment with lenvatinib is initiated earlier in more fit patients with progressive RR-DTC. In addition, the higher ORR and greater tumor shrinkage in patients with an ECOG PS of 0 suggest that the improvement in clinical benefit seen in these patients is not solely because of a better performance status.

Evidence regarding the prognostic nature of NLR for thyroid cancers is somewhat equivocal. A study assessing NLR in patients with DTC versus benign thyroid nodules found a correlation between NLR and tumor size, but no significant difference in NLR between cancer and control groups (26). Similarly, a meta-analysis indicated that there was no significant difference in NLR between patients with DTC and patients with benign nodules (mean difference = 0.19 [CI −0.09 to 0.46]) (27). A large meta-analysis across solid tumors, however, did find an association between high NLR and a shorter OS (HR 1.81 [CI 1.67–1.97]; *p* < 0.001) (19). In an exploratory analysis of NLR in a phase 2 trial of lenvatinib, a trend indicating that baseline NLR could affect PFS was seen, although statistical significance was not reached (28). Our exploratory analysis of data from the phase 3 SELECT provides additional evidence of a relationship between lower baseline NLR and longer PFS and OS in patients with RR-DTC that was treated with lenvatinib.

This analysis was limited by the *post hoc* nature of the assessment. In addition, concurrent corticosteroid treatment can increase NLR (29), creating a potential confounding factor for these data. However, only 20 patients (7.7%) in the lenvatinib treatment arm were taking corticosteroids at baseline, and corticosteroid treatment did not appear to influence outcomes by NLR status (Supplementary Table S2 and Supplementary Fig. S3). This study also had a potential selection bias regarding tumor size, and an imbalance between number of metastatic sites at baseline between ECOG PS groups. In addition, tumor size was measured by the assessment of target lesions using RECIST v1.1, which is a surrogate for, but not absolute measure of, total tumor burden. There was also a difference in rates of lenvatinib dose reduction and discontinuation between ECOG PS 0 and 1, which could impact efficacy and create bias.

Lead-time bias may also have impacted the OS results observed in this analysis. For example, it is possible that patients with lower baseline ECOG PS and NLR were diagnosed earlier in the course of their disease; such early detection could make OS appear improved compared with patients diagnosed at a later disease stage, irrespective of treatment. Notably, however, PFS and ORR (measures of efficacy not impacted by lead time bias) were also improved in patients with lower ECOG PS. A further limitation is that this study was not powered to assess the predictive validity of ECOG PS or NLR; therefore, additional studies are needed to determine whether any of these indicators are predictive for lenvatinib versus placebo. Finally, ECOG PS may be assessed inconsistently by clinicians and may be unreliable as a prognostic marker, particularly in a real-world setting (30).

Despite these limitations, this analysis indicates that correlates of efficacy, including ECOG PS and NLR, may provide prognostic value and treatment guidance in patients with RR-DTC. For clinicians, deciding on the optimal time to initiate tyrosine kinase inhibitor therapy can be challenging because of the desire to avoid AEs (31), but this analysis suggests that, in patients with RR-DTC, it may be advantageous to start lenvatinib treatment before ECOG PS and NLR worsen, and tumor size increases.

## Supplementary Material

Supplemental data

Supplemental data

Supplemental data

Supplemental data

Supplemental data

Supplemental data

Supplemental data

Supplemental data

Supplemental data
