# Supplementary material for: Correlation of Performance Status and Neutrophil-Lymphocyte Ratio with Efficacy in Radioiodine-Refractory Differentiated Thyroid Cancer Treated with Lenvatinib
Source: Thyroid. 2021 Aug 3;31(8):1226–34. doi: 10.1089/thy.2020.0779 (PMC8377516; doi:10.1089/thy.2020.0779)
Supplement: Supplemental data [file Supp_MaterialS1.docx]

**Supplemental Material**

**Supplemental Methods**

Patients were eligible for this study comparing lenvatinib versus placebo in patients with RR-DTC if they had progressive measurable RR-DTC, with ≤1 previous treatment with a tyrosine kinase inhibitor. Evidence of disease progression, according to Response Evaluation Criteria In Solid Tumors version 1.1 (RECIST v1.1) and confirmed by independent review, must have occurred within 13 months prior to informed consent. Other eligibility criteria included age ≥18 years and adequate organ function.

**Supplemental Results**

PFS was assessed according to baseline ECOG PS in patients who received placebo. While there was no statistically significant difference in PFS by baseline ECOG among patients randomly assigned to placebo, patients with a baseline ECOG PS of 0 had numerically better PFS than patients with a baseline ECOG PS of 1 (HR 0.69 [95% CI: 0.47–1.01]; *P*=0.0590) (**Supplemental** **Figure 4A**). The lack of significance is primarily due to the small sample size. Similarly, OS was assessed, and among patients randomly assigned to placebo, patients with a baseline ECOG PS of 0 had improved OS compared with patients with a baseline ECOG PS of 1 (HR 0.21 [95% CI: 0.11–0.41]; *P*<0.0001) (**Supplemental** **Figure 4B**).

PFS and OS were also assessed according to baseline NLR in patients who received placebo. PFS was prolonged in patients with an NLR ≤3 versus patients with an NLR >3 (HR 0.54 [95% CI: 0.37–0.79]; *P*=0.0016) (**Supplemental Figure 5A)**. OS was also prolonged in patients with an NLR ≤3 versus patients with an NLR >3 (HR 0.33 [95% CI: 0.17–0.63]; *P*=0.0005) (**Supplemental Figure 5B**).
