# Supplementary material for: Correlation of Performance Status and Neutrophil-Lymphocyte Ratio with Efficacy in Radioiodine-Refractory Differentiated Thyroid Cancer Treated with Lenvatinib
Source: Thyroid. 2021 Aug 3;31(8):1226–34. doi: 10.1089/thy.2020.0779 (PMC8377516; doi:10.1089/thy.2020.0779)
Supplement: Supplemental data [file Supp_TableS1.docx]

**Supplemental Table 1.** Baseline absolute neutrophil and lymphocyte counts by NLR in patients randomly assigned to receive lenvatinib

| **Parameter, mean (SD)** | **NLR ≤ 3**  **(n = 121)** | **NLR > 3**  **(n = 140)** |
| --- | --- | --- |
| **Absolute neutrophil count, x 10^9^/L** | 3.3 (1.0) | 5.3 (2.2) |
| **Absolute lymphocyte count x 10^9^/L** | 1.6 (0.6) | 1.0 (0.4) |

NLR, neutrophil-to-lymphocyte ratio; SD, standard deviation.
