# Supplementary material for: Correlation of Performance Status and Neutrophil-Lymphocyte Ratio with Efficacy in Radioiodine-Refractory Differentiated Thyroid Cancer Treated with Lenvatinib
Source: Thyroid. 2021 Aug 3;31(8):1226–34. doi: 10.1089/thy.2020.0779 (PMC8377516; doi:10.1089/thy.2020.0779)

**Supplemental Figure 1.** Baseline number of metastatic sites by NLR in patients randomly assigned to receive lenvatinib NLR, neutrophil-to-lymphocyte ratio.


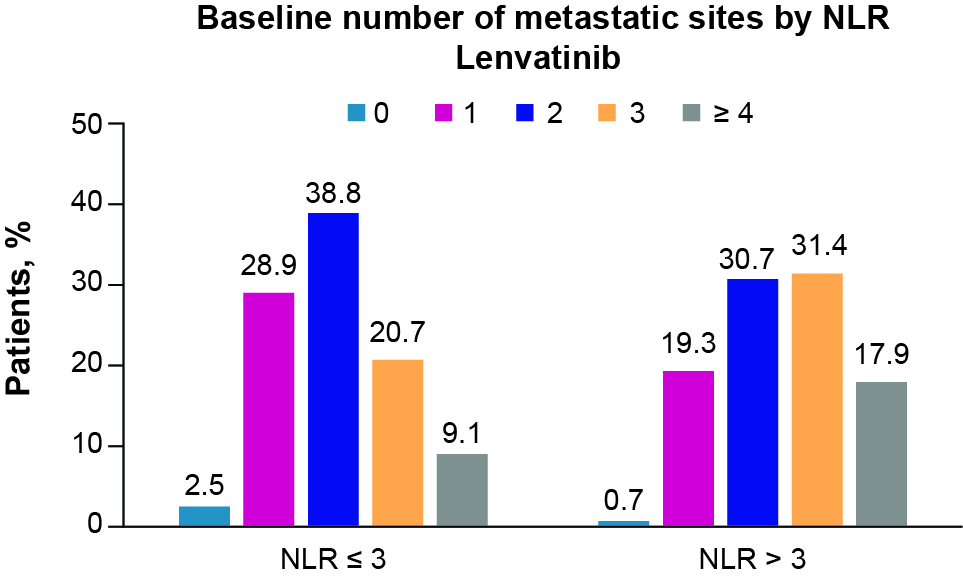

Supplement: Supplemental data [file Supp_FigureS1.docx]
