# Supplementary material for: Correlation of Performance Status and Neutrophil-Lymphocyte Ratio with Efficacy in Radioiodine-Refractory Differentiated Thyroid Cancer Treated with Lenvatinib
Source: Thyroid. 2021 Aug 3;31(8):1226–34. doi: 10.1089/thy.2020.0779 (PMC8377516; doi:10.1089/thy.2020.0779)

**Supplemental Figure 2.** Percentage changes in the sums of target lesions over time (from baseline to postbaseline nadir in A; from baseline to each study visit in B) in patients randomly assigned to receive lenvatinib, and with a baseline ECOG PS of 0 or 1 ECOG PS, Eastern Cooperative Oncology Group performance status.


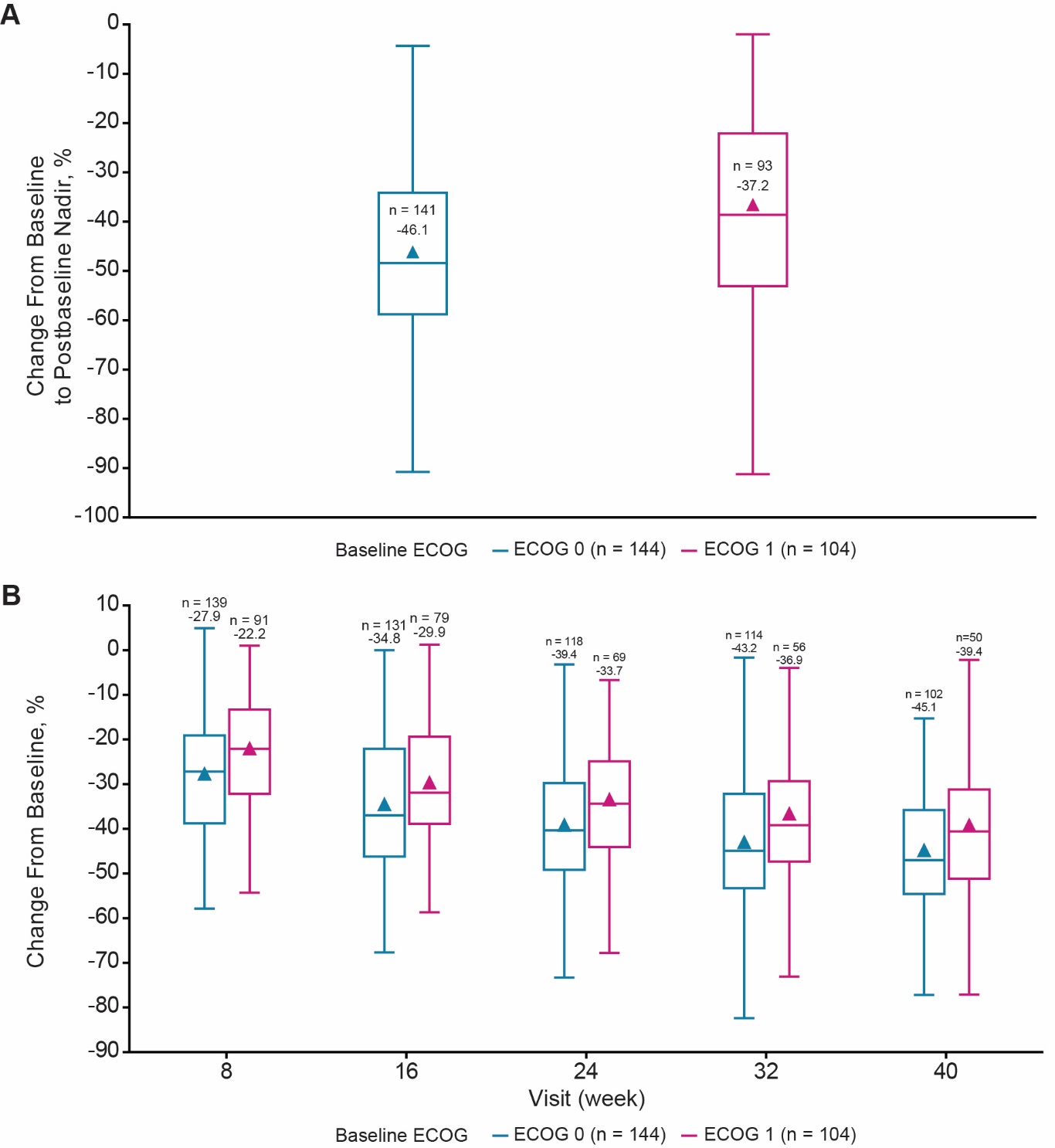

Supplement: Supplemental data [file Supp_FigureS2.docx]
