# Supplementary material for: Correlation of Performance Status and Neutrophil-Lymphocyte Ratio with Efficacy in Radioiodine-Refractory Differentiated Thyroid Cancer Treated with Lenvatinib
Source: Thyroid. 2021 Aug 3;31(8):1226–34. doi: 10.1089/thy.2020.0779 (PMC8377516; doi:10.1089/thy.2020.0779)

**Supplemental Figure 3.** Kaplan–Meier plots of PFS (A) and OS (B) by NLR in patients randomly assigned to received lenvatinib, and without concomitant steroid use at baseline OS and PFS medians were calculated by Kaplan–Meier estimates and the corresponding 95% CIs were calculated with a generalized Brookmeyer and Crowley method. Hazard ratios were estimated from an unstratified Cox proportional hazard model and *P*-values were based on an unstratified log-rank test.

CI, confidence interval; NE, not estimable; NLR, neutrophil-to-lymphocyte ratio; OS, overall survival; PFS, progression-free survival.


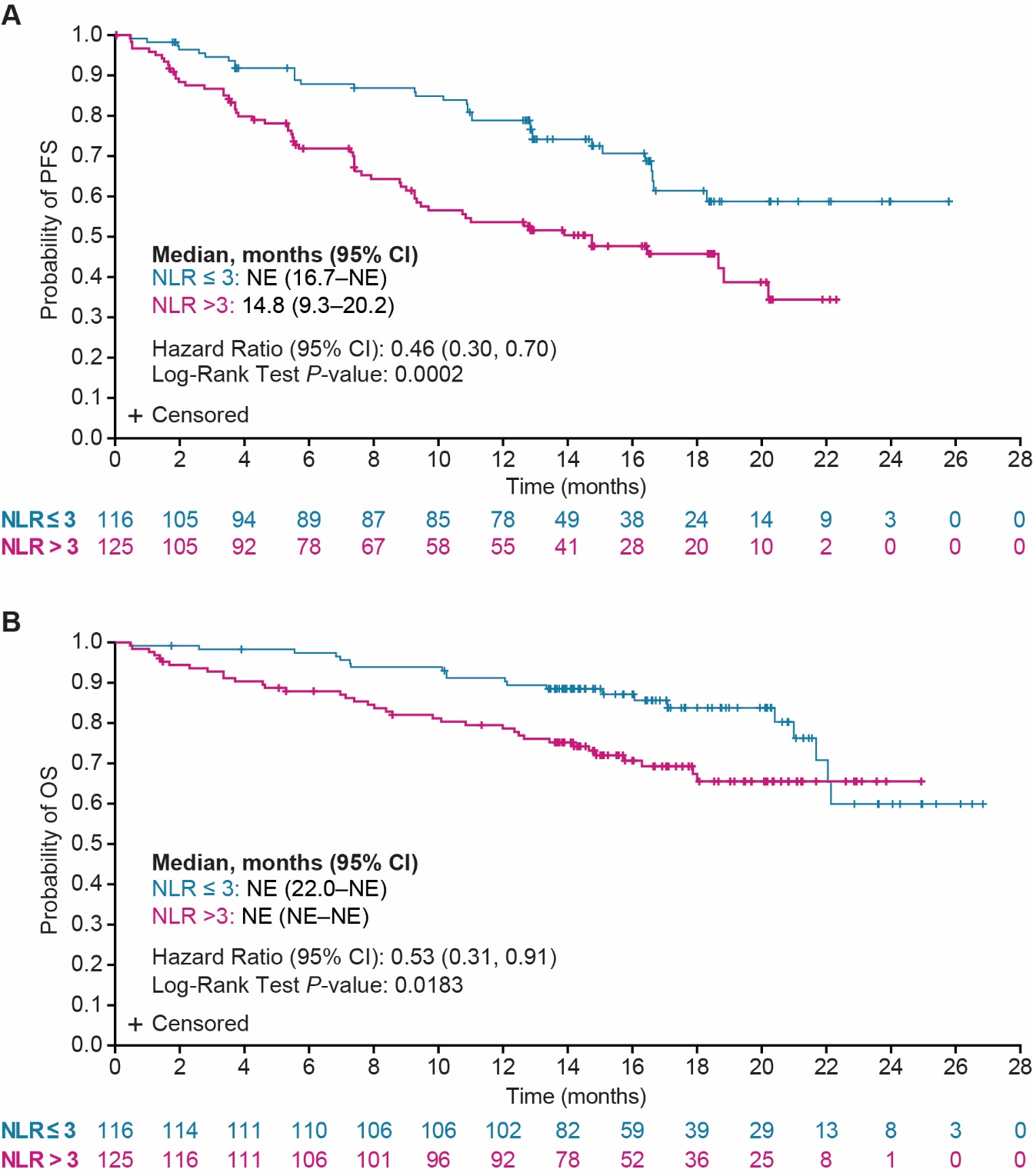

Supplement: Supplemental data [file Supp_FigureS3.docx]
