# Supplementary material for: Correlation of Performance Status and Neutrophil-Lymphocyte Ratio with Efficacy in Radioiodine-Refractory Differentiated Thyroid Cancer Treated with Lenvatinib
Source: Thyroid. 2021 Aug 3;31(8):1226–34. doi: 10.1089/thy.2020.0779 (PMC8377516; doi:10.1089/thy.2020.0779)
Supplement: Supplemental data [file Supp_TableS2.docx]

**Supplemental Table 2.** Summary of objective response rates by NLR in patients randomly assigned to receive lenvatinib, with and without concomitant steroid use at baseline

| **Parameter** | **Full analysis set** | | **Patients without concomitant steroid use** | |
| --- | --- | --- | --- | --- |
|  | **NLR ≤ 3**  **(n = 121)** | **NLR > 3**  **(n = 140)** | **NLR ≤ 3**  **(n = 116)** | **NLR > 3**  **(n = 125)** |
| **Objective response rate, n (%)** | 85 (70.2) | 84 (60.0) | 83 (71.6) | 79 (63.2) |
| 95% CI | 62.1–78.4 | 51.9–68.1 | 63.3–79.8 | 54.8–71.7 |
| **Odds ratio** | 1.57 | | 1.46 | |
| 95% CI | 0.94–2.64 | | 0.85–2.52 | |

CI, confidence interval; NLR, neutrophil-to-lymphocyte ratio; OR, odds ratio.
