# Supplementary material for: Correlation of Performance Status and Neutrophil-Lymphocyte Ratio with Efficacy in Radioiodine-Refractory Differentiated Thyroid Cancer Treated with Lenvatinib
Source: Thyroid. 2021 Aug 3;31(8):1226–34. doi: 10.1089/thy.2020.0779 (PMC8377516; doi:10.1089/thy.2020.0779)
Supplement: Supplemental data [file Supp_FigureS4.docx]

**Supplemental Figure 4.** Kaplan–Meier plots of PFS (A) and OS (B) by baseline ECOG PS in patients randomly assigned to receive placebo


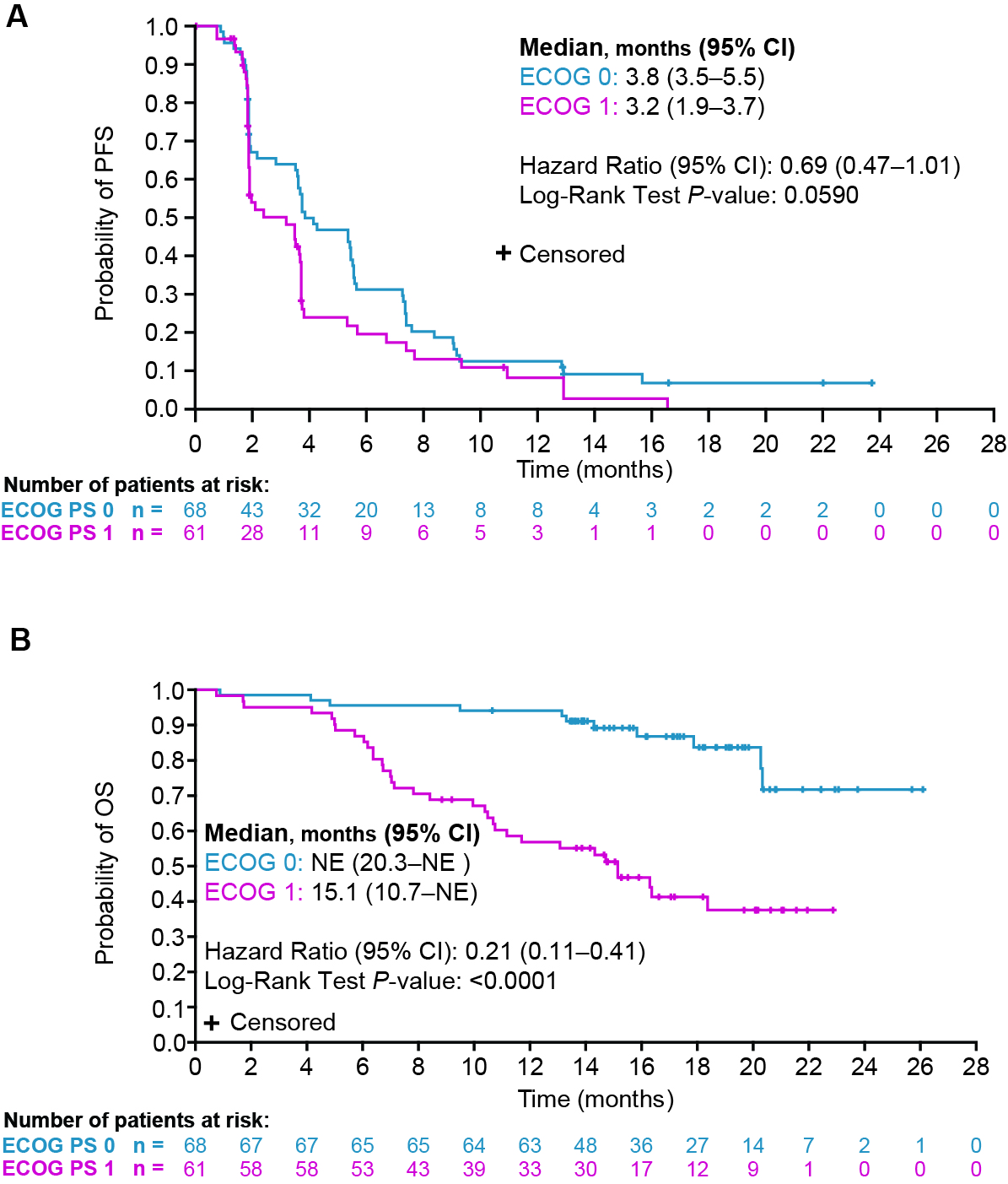


CI, confidence interval; ECOG PS, Eastern Cooperative Oncology Group performance status; NE, not estimable; OS, overall survival; PFS, progression-free survival.
