# Supplementary material for: Correlation of Performance Status and Neutrophil-Lymphocyte Ratio with Efficacy in Radioiodine-Refractory Differentiated Thyroid Cancer Treated with Lenvatinib
Source: Thyroid. 2021 Aug 3;31(8):1226–34. doi: 10.1089/thy.2020.0779 (PMC8377516; doi:10.1089/thy.2020.0779)
Supplement: Supplemental data [file Supp_TableS3.docx]

**Supplemental Table 3.** Overview of TEAEs by baseline ECOG PS (0 or 1) among patients randomly assigned to receive lenvatinib

| **Preferred term, n (%)** | **Baseline**  **ECOG PS 0**  **(n = 144)** | **Baseline**  **ECOG PS 1**  **(n = 104)** |
| --- | --- | --- |
| **Patients with any TEAE** | 144 (100.0) | 103 (99.0) |
| **Patients with any treatment-related TEAEs** | 142 (98.6) | 99 (95.2) |
| **Patients with treatment-related TEAEs, grade ≥3** | 112 (77.8) | 75 (72.1) |
| **Serious TEAEs**  Patients with any fatal serious TEAEs  Patients with any nonfatal serious TEAEs  With persistent or significant disability  That require or prolong hospitalization  Life-threatening  Important medical events | 59 (41.0)  6 (4.2)  58 (40.3)  3 (2.1)  53 (36.8)  4 (2.8)  8 (5.6) | 63 (60.6)  11 (10.6)  62 (59.6)  3 (2.9)  55 (52.9)  8 (7.7)  12 (11.5) |
| **Patients with**  TEAEs leading to study drug withdrawal  TEAEs leading to study drug dose reduction  TEAEs leading to study drug interruption | 16 (11.1)  106 (73.6)  118 (81.9) | 20 (19.2)  62 (59.6)  86 (82.7) |
| **Patients with any TEAEs**  Diarrhea  Hypertension  Decreased appetite  Weight decreased  Nausea  Fatigue  Headache  Stomatitis  Vomiting  Palmar-plantar erythrodysesthesia syndrome  Dysphonia  Arthralgia  Constipation  Proteinuria  Asthenia  Cough  Dry mouth  Rash | 144 (100.0)  110 (76.4)  104 (72.2)  78 (54.2)  78 (54.2)  67 (46.5)  66 (45.8)  60 (41.7)  58 (40.3)  53 (36.8)  52 (36.1)  51 (35.4)  44 (30.6)  42 (29.2)  40 (27.8)  38 (26.4)  36 (25.0)  29 (20.1)  25 (17.4) | 103 (99.0)  56 (53.8)  69 (66.3)  54 (51.9)  49 (47.1)  47 (45.2)  39 (37.5)  37 (35.6)  29 (27.9)  34 (32.7)  29 (27.9)  27 (26.0)  21 (20.2)  30 (28.8)  38 (36.5)  24 (23.1)  20 (19.2)  12 (11.5)  22 (21.2) |

ECOG PS, Eastern Cooperative Oncology Group performance status; TEAE, treatment-emergent adverse event.
